# Downbeat nystagmus in association with the dorsal midbrain syndrome: proposed mechanisms, literature review, and case series

**DOI:** 10.3389/fneur.2025.1716511

**Published:** 2025-12-09

**Authors:** Lea Saab, Eric R. Eggenberger, Catherine Cho, Maximilian U. Friedrich, Janet C. Rucker

**Affiliations:** 1 Department of Neurology, NYU Grossman School of Medicine, New York, NY, United States; 2 Department of Ophthalmology, Mayo Clinic, Jacksonville, FL, United States; 3 Department of Neurology, Mayo Clinic, Jacksonville, FL, United States; 4 Department of Neurosurgery, Mayo Clinic, Jacksonville, FL, United States; 5 Department Otolaryngology-Head and Neck Surgery, NYU Grossman School of Medicine, New York, NY, United States; 6 Department of Neurology, University Hospital Ulm, Ulm, Germany; 7 Department of Ophthalmology, NYU Grossman School of Medicine, New York, NY, United States

**Keywords:** downbeat nystagmus, interstitial nucleus of Cajal, dorsal midbrain, Parinaud syndrome, posterior commissure

## Abstract

**Introduction:**

Downbeat nystagmus (DBN) is classically attributed to cerebellar pathology. Far less often, DBN arises from brainstem disease, with midbrain etiologies being exceptionally rare and poorly characterized. In particular, DBN with the dorsal midbrain syndrome has been sporadically reported and its mechanism is unclear.

**Methods/results:**

We analyzed non-human primate and human literature on vertical gaze and brainstem-related DBN in the context of three patients with DBN and dorsal midbrain syndrome. Upgaze paresis was universal, skew deviation present in two, and parkinsonism developed in two after shunt-related complications. We reviewed the literature using strict criteria requiring DBN in central gaze and at least two definitive features of the dorsal midbrain syndrome, identifying two additional patients.

**Discussion:**

In five patients with DBN and dorsal midbrain syndrome, aqueductal stenosis or compression with upgaze paresis were unifying features. Potential mechanisms for DBN include involvement of the interstitial nucleus of Cajal, disruption of descending midbrain projections to paramedian tracts, or unstable cerebellar outflow, but clinical and experimental evidence makes these explanations less compelling. Converging evidence from our series, prior reports, and non-human primate studies suggests bilateral posterior commissural dysfunction related to aqueductal pathology as the most plausible mechanism for DBN with the dorsal midbrain syndrome.

## 1 Introduction

Downbeat nystagmus (DBN) is a spontaneous vertical jerk nystagmus characterized by upward slow phases and downward corrective quick phases. It is most often due to dysfunction of the vestibulocerebellar flocculus and paraflocculus or nodulus and uvula, and several mechanisms have been proposed. First, the cerebellar control of vertical eye movements is asymmetric for upward versus downward movements; for example, the cerebellum inhibits upward, but not downward, eye movements (1, 2) and floccular Purkinje cells predominantly encode downward movements (3). Cerebellar dysfunction, thus, leads to unopposed upward drifts of the eyes and DBN. Second, dysfunction of the nodulus and uvula, which integrate otolith-driven translational vestibulo-ocular signals that stabilize gaze during linear motion (4), disrupts gravity-sensitive vertical tone and produces DBN (5). Third, instability of vertical neural integration due to poor cerebellar feedback control is proposed as the etiology for DBN with increasing or variable velocity slow phases (6, 7).

Very rarely, DBN occurs with brainstem lesions in the medulla or as a component of internuclear ophthalmoplegia (INO) due to medial longitudinal fasciculus lesions. With medullary lesions, the paramedian tracts that have excitatory projections to the cerebellar flocculus are implicated (8). DBN with INO likely results from dysfunction of excitatory posterior canal projections to eye muscles for downward gaze (9, 10), Pure DBN usually occurs with bilateral INO from bilateral medial longitudinal fasciculus lesions (11), whereas unilateral lesions more often cause combined torsional-vertical nystagmus (12).

DBN from midbrain lesions is exceedingly uncommon, representing only 2% of DBN cases (13), and the combination of DBN with the dorsal midbrain syndrome has received little attention. We review the literature on this unusual combination, describe three additional patients with DBN in the setting of midbrain pathology as evidenced by components of the dorsal midbrain syndrome, and explore potential mechanisms by which midbrain structures involved with vertical eye movement control may produce DBN.

## 2 Methodology of literature review

In depth literature review was undertaken to identify previously published cases of DBN in association with the dorsal midbrain syndrome. A focused literature search in PubMed from database inception through 2025 was performed to identify reports of DBN associated with midbrain pathology. Initial search terms included downbeat nystagmus and midbrain or mesencephalon, with sensitivity expansions incorporating relevant anatomic terms such as interstitial nucleus of Cajal, posterior commissure, pretectal. No study design filters were applied at the search stage. Titles and abstracts were screened independently by two reviewers, with full texts retrieved for potentially eligible reports. Backward and forward citations were collected from report bibliographies from included papers to capture additional cases. Manuscripts reviewed varied in quality, but all pertinent cases of combined DBN and dorsal midbrain syndrome contained enough detail to be included. Medications were not included in most manuscripts, so effects of medications could not be ascertained. Cases with potential secondary causes of DBN were excluded. Cases of mixed torsional-vertical nystagmus were not included. Only cases with pure DBN present in central gaze in both eyes were included. At least two of the core clinical features of the dorsal midbrain syndrome had to be present for inclusion. Core clinical features meeting criteria included upgaze paresis, pupillary light-near dissociation, eyelid retraction, and convergence-retraction nystagmus. Cases with INO on examination or cerebellar pathology present on imaging were excluded, as DBN in those cases might be attributable, respectively, to medial longitudinal fasciculus or cerebellar pathology.

## 3 Results: case descriptions

### 3.1 Case 1

**Figure 1:**
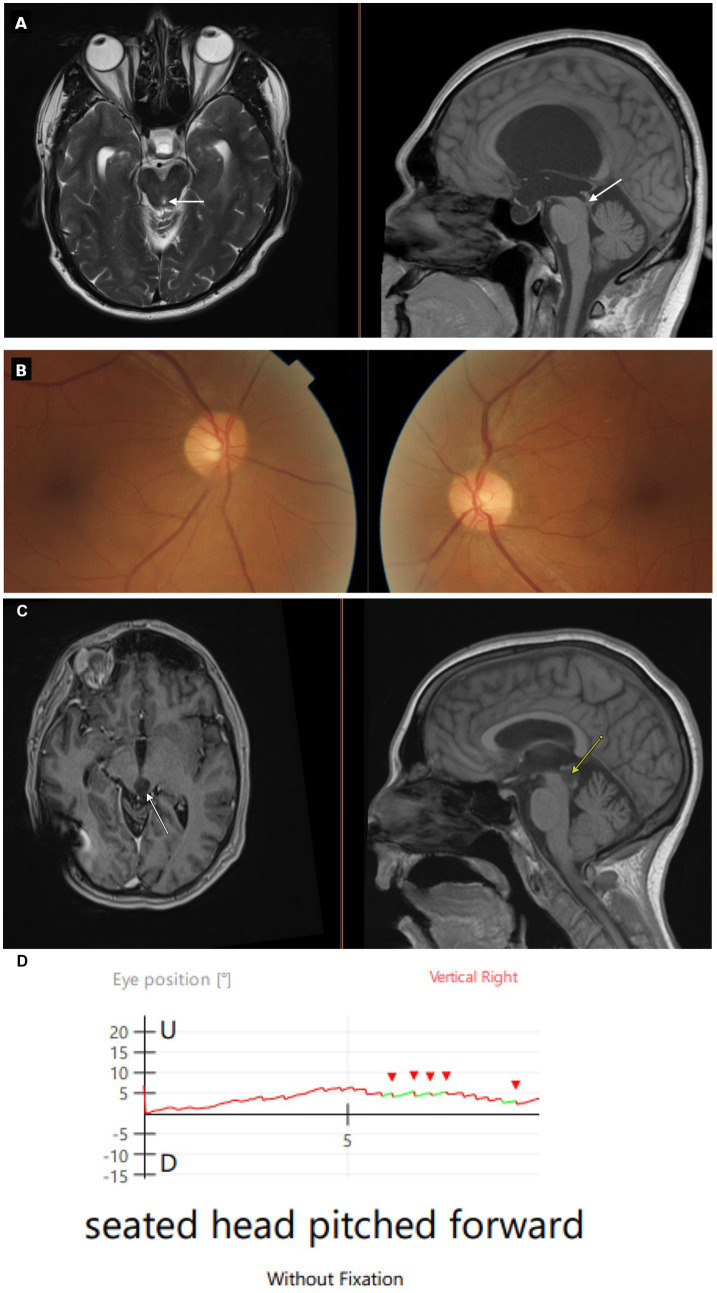
Clinical data. **(A)** Case 1 – MRI (axial T2-weighted left, sagittal T1-weighted right) demonstrates marked third and lateral ventricular dilatation, normal fourth ventricle caliber, and distal cerebral aqueduct narrowing (arrows). **(B)** Case 1 – Fundus photographs showing intorsion of the left eye (right side of photograph) and extorsion of the right eye (left side of photograph). **(C)** Case 2 – MRI (axial T1-weighted left, sagittal T1-weighted right) shows a non-enhancing tectal lesion involving the quadrigeminal plate to the left of the midline, with cerebral aqueduct compression. **(D)** Case 2 – Vertical eye position during head pitch forward (no fixation). Right-eye trace shows upward slow-phase drift with corrective downward quick phases (red carets).

A 58-year-old man underwent brain MRI for progressive gait impairment. MRI revealed obstructive hydrocephalus due to aqueductal stenosis (Figure 1A). He underwent ventriculoperitoneal shunt placement, complicated by recurrent subdural hematomas requiring multiple surgical evacuations. After shunt placement, he reported persistent binocular vertical diplopia. He denied oscillopsia, headaches, or pulsatile tinnitus. Ultimately, he underwent endoscopic third ventriculostomy with shunt removal. He later developed new-onset parkinsonism, characterized by facial masking with left arm tremor and bradykinesia, along with ocular motor findings consistent with rostral midbrain involvement. Both were attributed to mechanical distortion of the rostral midbrain. The parkinsonism, more specifically, was attributed to functional disconnection of nigrostriatal pathways.

Neuro-ophthalmic exam revealed partial dorsal midbrain syndrome with pupillary light-near dissociation, eyelid retraction, and severe upgaze limitation (<10% of normal). Convergence was impaired. No convergence-retraction nystagmus was elicited. Vertical saccades were markedly slow and optokinetic quick phases were absent in both vertical directions; horizontal saccades were normal with preserved optokinetic quick phases. DBN was present in lateral downgaze while the patient fixated a visual target and visible in central gaze on fundoscopy with the contralateral eye fixating a visual target. A skew deviation was present, with a small angle left hypertropia, intorsion of the left eye and extorsion of the right eye (Figure 1B). There was no papilledema or optic atrophy. No further cerebellar signs were present.

### 3.2 Case 2

A 43-year-old woman was incidentally discovered on brain MRI to have an 8 × 8 mm non-enhancing left quadrigeminal plate mass, consistent with a low-grade tectal glioma causing aqueductal compression. Two years later, the mass had grown to 13 × 13 mm (Figure 1C) and she developed acute hydrocephalus with right leg numbness and progressive encephalopathy. She underwent ventriculoperitoneal shunt placement, followed by multiple revisions for persistent dysfunction, characterized by hypersomnolence, catatonia, and parkinsonism. Her condition gradually improved following a third ventriculostomy. After surgical stabilization, she developed persistent dizziness.

Neuro-ophthalmic exam revealed a partial dorsal midbrain syndrome with pupillary light-near dissociation, mild left eyelid retraction, and mild upgaze limitation (80% of normal). Convergence was impaired. No convergence-retraction nystagmus was elicited. DBN, while the patient fixated a visual target, was present with small amplitude in central gaze and increased in downgaze, right gaze, and left gaze, with maximal amplitude in downgaze (Supplementary Video S1). Upbeat nystagmus was present in upgaze. A skew deviation was present with a small angle comitant left hypertropia. There was no papilledema or optic atrophy. No further cerebellar signs were present. Several months after the above exam, DBN was visible on high-resolution infrared video with visual fixation removed and the head pitched forward. Position traces demonstrated constant velocity slow phases of 2 deg./s (Figure 1D; Supplementary Video S1).

### 3.3 Case 3

A 35-year-old woman presented with constitutional symptoms, including low grade fevers, followed by sequential cranial neuropathies involving complete bilateral hearing loss, a transient partial right sixth nerve palsy, a persistent right seventh nerve palsy, and severe headaches with meningismus. Cerebrospinal fluid revealed pleocytosis and brain MRI demonstrated diffuse leptomeningeal enhancement along the basal cisterns and cerebral convexities, with middle ear biopsy detecting aspergillus that was treated with antifungal medication. The course was complicated by progressive obstructive hydrocephalus on serial imaging, most pronounced at the third and lateral ventricles with relative sparing of the fourth ventricle, consistent with aqueductal stenosis. She underwent ventriculoperitoneal shunt placement. Episodes of intracranial hypotension temporarily exacerbated symptoms before responding to pressure-directed interventions.

Neuro-ophthalmic evaluation revealed partial dorsal midbrain syndrome with complete upgaze palsy with intermittent convergence-retraction nystagmus on upward saccade attempts. Pupillary light-near dissociation and eyelid retraction were absent. Complete downgaze palsy was also present. Prominent DBN, while the patient fixated a visual target, was present in central gaze and increased in lateral downgaze.

## 4 Discussion

### 4.1 DBN in association with the dorsal midbrain syndrome

The combination of DBN in association with the dorsal midbrain syndrome is exceedingly rare. The dorsal midbrain syndrome is a well-defined entity that localizes to the posterior commissure in the dorsal midbrain (14). It is also referred to as Parinaud syndrome, the Sylvian aqueduct syndrome, and the pretectal syndrome. Classic etiologies include pineal tumors and hydrocephalus, though metastatic disease, demyelination, infarction or infection such as tuberculoma or neurocysticercosis can also be causative (15–19). The core exam features include upgaze paresis, pupillary light-near dissociation, eyelid retraction (also called Collier’s sign), and convergence-retraction nystagmus; however, not all patients manifest all four core features (18). Downgaze palsy and skew deviation are also often present, and were observed in our patients. It is unclear if these findings result directly from the posterior commissure lesion or from simultaneous involvement of adjacent structures, such as the rostral interstitial medial longitudinal fasciculus, which contains excitatory burst neurons that generate vertical and torsional saccades, or the interstitial nucleus of Cajal, which is the neural integrator for vertical gaze holding.

**Figure 2:**
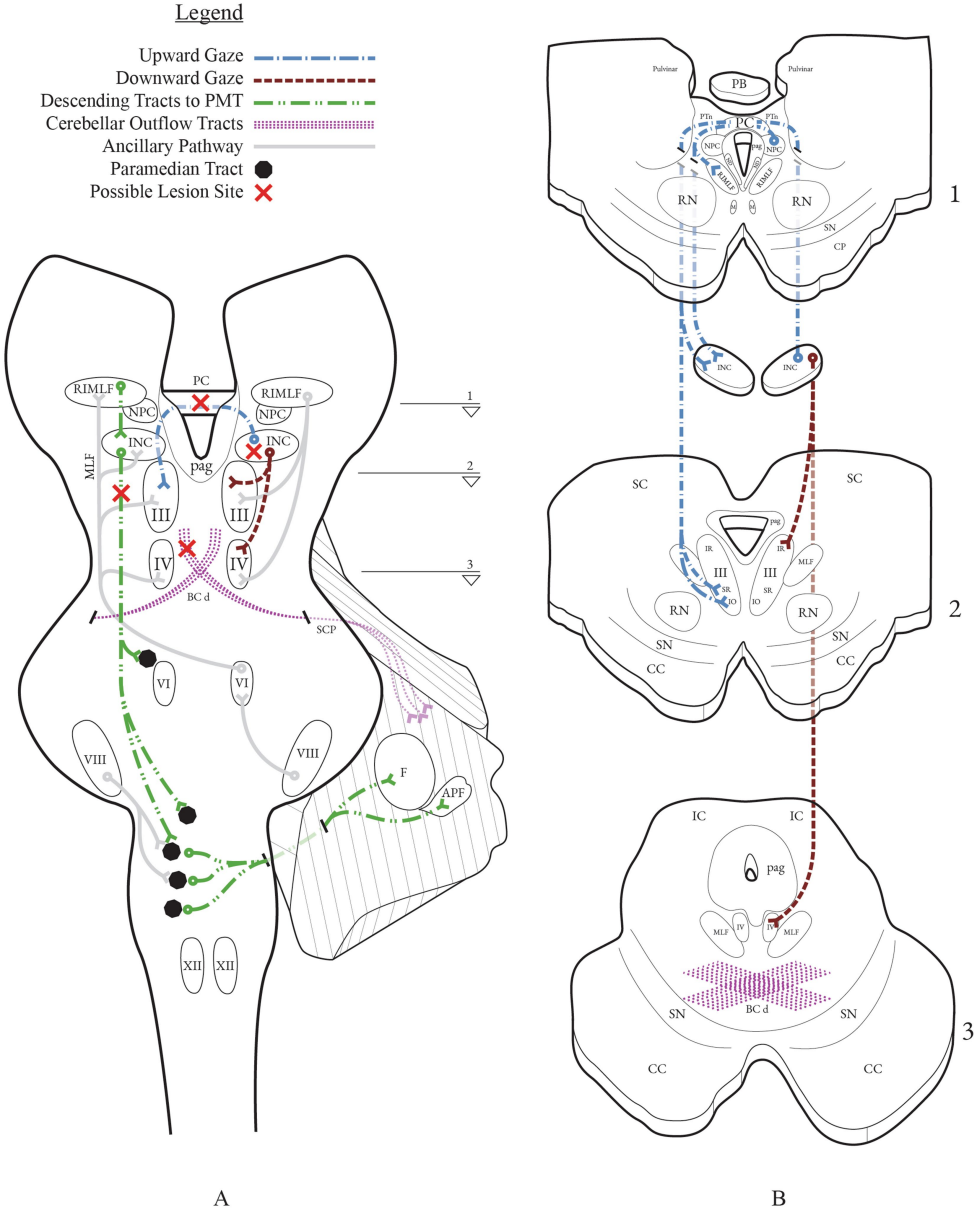
Vertical gaze networks with possible lesion sites implicated in downbeat nystagmus from midbrain lesions. **(A)** Coronal section through the brainstem depicting the principal vertical gaze centers and their projections: RIMLF (burst generator), INC (neural integrator), NPC, PC, and ocular motor nuclei (III, IV). Descending projections from the INC to PMT cell groups (black circles; green dashed line) in the pons and relay to the flocculus and accessory paraflocculus. Cerebellar outflow courses through the BC/SCP and its decussation (BCd) in the caudal midbrain (purple stipple). **(B)** Axial panels B1–B3 correspond to the slice levels 1–3 indicated on **(A)**. Upgaze signals from INC cross in the PC to the contralateral SR and IO subnuclei of III; downgaze projections run predominantly ipsilaterally to the IR subnucleus of III and to IV (SO). This laterality makes PC lesions more disruptive to upgaze than downgaze, biasing vertical balance toward a downbeat drift.

The upgaze paresis in the dorsal midbrain syndrome is attributed to the fact that signals for generation of upward eye movements from the interstitial nucleus of Cajal project to motoneurons for upward eye movements (superior rectus and inferior oblique) through the posterior commissure. In contrast, signals for generation of downward eye movements project from the interstitial nucleus of Cajal, not only through the posterior commissure, but also ipsilaterally directly to the motoneurons for downward eye movement (inferior rectus and superior oblique) (Figure 2B) (20). Thus, posterior commissure lesions cause upgaze paresis with relatively spared downgaze (i.e., normal or with slowing of downward saccades).

True nystagmus with pathologic slow drift of the eyes followed by quick resetting phases is not typical in the dorsal midbrain syndrome. Though named convergence-retraction nystagmus, this core dorsal midbrain syndrome feature is generally accepted to be a disorder of saccades or the vergence system, rather than true nystagmus as there is no initiating slow phase (21, 22). Very few fully-described individual cases of the dorsal midbrain syndrome in association with DBN have been reported (Table 1). In two large series of patients with dorsal midbrain syndrome, DBN was reported in a small proportion of patients, but individual patient data was not provided, so conclusions cannot be drawn from those studies as to whether lesions were restricted to the posterior commissure and with which etiologies or other examination findings DBN might be associated. The first of these studies included 206 patients with the dorsal midbrain syndrome; DBN in central position was identified in 3.8% of patients (18). In the second study, DBN was reported in 8% of 40 patients; however, this study does not report whether DBN was present in central gaze or only elicited in eccentric gaze (23).

**Table 1:** Clinical, neuro-ophthalmologic, and imaging characteristics of dorsal midbrain lesions presenting with downbeat nystagmus.

Author (Year)	Age/Sex	Etiology	Imaging or postmortem studies	Limited upgaze	Pupillary light near dissociation	Eyelid retraction	Convergence-retraction nystagmus	Convergence absent	Skew deviation	Cerebellar signs	Other brainstem findings
Case 1 – current series	58/M	Aqueductal stenosis, hydrocephalus	MRI with marked third and lateral ventricular dilatation, normal fourth ventricle caliber	Y	Y	Y	N	Y	Y	N	Parkinsonism
Case 2 – current series	43/F	Tectal glioma, aqueductal compression, hydrocephalus	MRI with 8 × 8 mm tectal glioma, third and lateral ventricular dilatation, normal fourth ventricle caliber	Y	Y	Y–L	N	Y	Y	N	Parkinsonism
Case 3 – current series	75/M	Infectious meningoencephalitis, aqueductal stenosis, hydrocephalus	MRI with leptomeningeal enhancement at skull base, third and lateral ventricular dilatation, normal fourth ventricle caliber	Y	N	N	Y	?	N	N	Bilateral hearing loss, right-sided facial weakness
Keane 1976 (19)	46/M	Metastatic melanoma	Neuropathology tumor almost completely replaced the PC, partially involved the pineal body, and formed a layer over the inferior posterior third ventricle and proximal aqueduct floor, with Wallerian degeneration of residual posterior commissure fibers	Y	Y	Y	Y	Y	Y	?	N
Barrer 1980 (53)	19/F	Aqueductal stenosis, hydrocephalus	CT scan with dilated lateral and third ventricle	Y	Y	Y	?	?	N	N	Upper motor neuron – all 4 extremities, stupor, parkinsonism

Data are shown for three patients in the current series and two historical cases. Y, present; N, absent; ?, not reported; L, left.

Two patients were identified in the literature, in addition to our three patients, who met our criteria of at least two definitive core features of the dorsal midbrain syndrome and DBN in central gaze (Table 1). Of these five patients, all five had upgaze paresis, four had pupillary light-near dissociation and eyelid retraction, and two had convergence-retraction nystagmus. Etiologies included congenital hydrocephalus in two and, in one each, midbrain tectal glioma, infectious meningoencephalitis, and metastatic disease. What they all had in common, evidenced either by neuroimaging or autopsy neuropathology, was aqueductal involvement via stenosis, compression, or direct tumor deposits. Two additional published cases that did not meet our criteria of at least two components of the dorsal midbrain syndrome are of interest, though not included in Table 1. The first of these developed DBN and alternating skew deviation on lateral gaze in the setting of decompensated congenital aqueductal stenosis (24). The second developed DBN upon attempted downgaze in association with gaze-evoked nystagmus, upgaze paresis, and slow vertical saccades below the horizontal meridian but without additional dorsal midbrain syndrome core features (25). Autopsy in this second patient after death following a midbrain infarct revealed compression and displacement of the cerebral aqueduct and posterior commissure.

The unifying feature of aqueductal pathology in all of our identified cases is notable. Hydrocephalus is a common cause of the dorsal midbrain syndrome (26), but in the literature, it is often not specified whether hydrocephalus is diffuse and communicating or obstructive in association with aqueductal stenosis (27). There is no universally accepted mechanistic theory regarding how hydrocephalus causes the dorsal midbrain syndrome. Various mechanisms have been proposed, including dilation of the cerebral aqueduct and third ventricle or the suprapineal recess or through direct compression or distortion of the posterior commissure (28). In contrast, in our cases, the aqueduct was narrowed by disease and this raises a question of whether aqueductal pathology may be a prerequisite for development of DBN in association with the dorsal midbrain syndrome. Interestingly, three of the five patients in our series, two with congenital and one with acquired aqueductal stenosis, also developed acute parkinsonism suggesting nigrostriatal injury, which has been previously reported (29). A recent review emphasized that midbrain distortion or periaqueductal compression can induce parkinsonism by functionally disconnecting these circuits, even without direct nigral injury (30).

Prior reports of shunt-treated adults with non-obstructive communicating hydrocephalus in whom shunt overdrainage led to dorsal midbrain syndrome, at times with parkinsonism, provide further support that aqueductal pathology may be causative (31, 32). It has been postulated that overshunting leads to secondary cerebral aqueductal stenosis that causes upward midbrain herniation against the tentorial notch and bulging of the third ventricle. Indeed, there was evidence of overdrainage after shunt placement in all three of our patients that may have exacerbated the pre-existing aqueductal stenosis: recurrent subdural hematomas in case 1, clinical deterioration in case 2, episodes of intracranial hypotension in case 3. As an alternative to overdrainage, a study of 28 patients with dorsal midbrain syndrome in the setting of shunt failure proposed that aqueductal stenosis produces a transtentorial pressure gradient that leads to midbrain dysfunction and may be relieved by shunt revision or, more often, by 3^rd^ ventriculostomy (33). Such a mechanism would more readily explain the case of DBN and dorsal midbrain syndrome with metastasis-related aqueductal stenosis, but without known hydrocephalus (19). These alternative mechanisms to raised intracranial pressure would also explain why none of our patients developed papilledema.

### 4.2 Potential mechanisms of DBN in association with the dorsal midbrain syndrome

Mechanisms of DBN in association with the dorsal midbrain syndrome are poorly understood. Consideration can be given to several hypothetical possibilities (Figure 2). Involvement of structures adjacent to the posterior commissure must be considered, including the interstitial nucleus of Cajal or descending projections from the midbrain to the lower brainstem paramedian tracts. A second possibility is unstable neural integration or vestibular signal impairment due to dysfunction of cerebellar outflow tracts in the midbrain. Lastly, and perhaps most plausibly, DBN could arise directly from the posterior commissure lesion causing the dorsal midbrain syndrome. We will discuss each of these possibilities in order of our opinion on their likelihood, starting with the least likely explanation.

#### 4.2.1 Could DBN arise from the interstitial nucleus of Cajal?

Stable gaze requires neural integration of velocity-encoded ocular motor commands into appropriate tonic eye movement position signals. The interstitial nucleus of Cajal is the neural integrator for vertical and torsional eye movements (34, 35). It receives afferent input from vestibular nucleus projections, saccadic burst neurons in the rostral interstitial medial longitudinal fasciculus, and pursuit pathways. It projects to the ocular motor neurons ipsilaterally and also through the posterior commissure (36, 37). Nystagmus can arise from lesions in this structure from both neural integrator failure and from vestibular asymmetries (35). Unilateral lesions of the interstitial nucleus of Cajal cause impaired vertical and torsional gaze holding, an ocular tilt reaction, and torsional-vertical (either upward or downward beating) nystagmus with ipsilesional torsional quick phases (35, 38, 39). Bilateral lesional effects in non-human primates restrict the vertical range of all eye movements, impair vertical vestibulo-ocular reflex gains, cause impaired gaze holding after all vertical and torsional movements, and cause vertical nystagmus (35). Vertical gaze-evoked nystagmus (upbeat in upgaze and downbeat in down gaze) occurred, as did upbeat nystagmus in central gaze with decreasing slow phase velocity suggesting neural integrator failure – but DBN in central gaze did not occur in these non-human primate studies. Thus, it seems unlikely that the interstitial nucleus of Cajal, itself, is responsible for DBN in association with the dorsal midbrain syndrome.

#### 4.2.2 Could DBN arise from descending midbrain projections to the paramedian tracts?

For vertical and torsional eye movements, as noted above, the neural integrator is the interstitial nucleus of Cajal. Normal function of ocular motor integration to maintain stable eye position requires interaction between the brainstem and cerebellum (40). The paramedian tracts, cell groups in the pontine and medullary midline tegmentum, facilitate such interactions. One of these paramedian tract cell groups, the nucleus pararaphales, receives signals from the vestibular nuclei related to upward eye movements and projects to the cerebellar flocculus and ventral paraflocculus (41–43). The interstitial nucleus of Cajal sends afferent signals to nucleus pararaphales (43). Inactivation of these paramedian tract cell groups in non-human primates leads to deficits similar to those created by cerebellar flocculus dysfunction: DBN and leaky neural integration with resultant gaze-evoked nystagmus (41). Given the proximity of the interstitial nucleus of Cajal to the posterior commissure, it is theoretically feasible that DBN in association with the dorsal midbrain syndrome could arise from a lesional effect in descending projections from the interstitial nucleus of Cajal to the paramedian tracts.

#### 4.2.3 Could DBN arise from unstable neural integration at the level of cerebellar outflow tracts?

As noted above, normal function of ocular motor neural integration requires input from the cerebellum (40). In addition, cerebellar signals from the flocculus and paraflocculus contribute to the angular vestibulo-ocular reflex (aVOR) by modulating semicircular canal signals, selectively suppressing anterior canal-driven upward eye velocity (1, 2). Loss of this inhibition permits upward drift and leads to DBN. Cerebellar signals from the nodulus and uvula contribute to the translational vestibulo-ocular reflex (tVOR) (4). Nodulus and uvula dysfunction have been associated with downbeat nystagmus in non-human primates and vertical asymmetry in the tVOR has been proposed as one explanation for idiopathic DBN (5, 44).

All of the above signals from the cerebellum have efferent projections through the brachium conjunctivum (superior cerebellar peduncle) that decussate after entry into the midbrain. The decussation, itself, is located in the caudal midbrain and would be unlikely to be affected in isolated rostral midbrain lesions, yet, from the decussation, these signals ascend to ocular motor structures in the midbrain and descend to ocular motor and vestibular structures in the lower brainstem. However, their direct pathway of projection within the rostral midbrain is not fully delineated. Dysfunction of ascending VOR signals in midbrain structures could be the cause of DBN with linear slow phase velocities. Alternately, cerebellar dysfunction may cause neural integrator output through the cerebellar feedback loop to decrease, creating leaky neural integration, or to increase, creating unstable neural integration. These deficits generate nystagmus with decreasing slow-phase velocity with leaky integration and increasing or variable slow-phase velocity with unstable integration (45, 46). Increasing velocity slow phase DBN is reported with several cerebellar pathologies, including paraneoplastic disease and Chiari malformations, and in flocculectomized non-human primates (6, 7, 47). One published case of interest in this mechanistic category of impaired cerebellar outflow as a midbrain cause for DBN had DBN and alternating skew deviation on lateral gaze in the setting of decompensated congenital cerebral aqueductal stenosis (24). The authors speculated that the DBN arose due to dysfunction at the level of the midbrain, though there was no definitive proof of this. The DBN in this patient had increasing velocity slow phases, suggesting unstable neural integration. This patient also had an alternating skew deviation, which deserves further attention.

Interestingly, in the largest published series of patients with the dorsal midbrain syndrome, 38% of 206 patients had a skew deviation and one-third of these had an alternating skew deviation on lateral gaze, in which the right eye elevates upon right gaze and the left eye elevates upon left gaze (18). In the largest published series of patients with alternating skew deviation, 27 of 47 patients had pretectal lesions, some also with DBN – though it was unclear if the DBN occurred in patients with pretectal or cerebellar lesions (48). Alternating skew deviation is classic ‘cerebellar eye sign’, likely due to abnormalities in otolith projections (49). It, thus, appears plausible that DBN could theoretically result at the midbrain level from dysfunction of otolithic cerebellar outflow tracts, though DBN due to otholithic dysfunction in non-human primates occurs in darkness and is suppressed by visual fixation (50). DBN was present with visual fixation in all of our patients. Further work is needed to understand the anatomic connections and trajectories of cerebellar outflow signals once they enter the brainstem through the brachium conjunctivum. None of our patients had alternating skew deviation and slow phase velocities appeared linear in the patient for whom we have eye tracking traces.

#### 4.2.4 Could DBN arise from the posterior commissure?

The most plausible hypothesis to explain DBN in our patients is that it arises directly from the posterior commissure. Four of ten non-human primates with surgically-created pretectal lesions had DBN in central gaze (51). All ten demonstrated components of the dorsal midbrain syndrome, but the only core feature consistently present in those with DBN was upgaze paresis. This was also true in all patients in our series. In this study, careful anatomic assessment of involvement of adjacent structures, such as the nucleus of the posterior commissure, the pretectum, and the interstitial nucleus of Cajal was undertaken. The only consistent anatomic region affected in those with DBN was bilateral involvement of the posterior commissure (52). Unfortunately, no mention of the anatomic appearance of the cerebral aqueduct was made. Vertical vestibular responses to bilateral caloric stimulation were significantly impaired, suggesting that DBN may arise from asymmetric dysfunction of posterior and anterior canal inputs projecting through the posterior commissure. However, in the oculographic traces, the DBN appeared to have increasing velocity slow phases, supporting unstable neural integration as the mechanism of DBN in these non-human primates with posterior commissural lesions. Thus, the nystagmus in this study still differs from that seen in our patients, at least for the single patient for whom we have oculographic traces.

It is well known that the interstitial nucleus of Cajal and nucleus of the posterior commissure project to the posterior commissure and that posterior commissural lesions decouple tonic positional signals from the ocular motoneurons. However, the precise pathway and distribution of vestibular, saccadic, pursuit, pupillary, and eyelid signals within the posterior commissure is not known. Nor is it known how or if the posterior commissure interfaces with information arising in the cerebellum. Despite this, the most plausible anatomic localization for DBN in association with the dorsal midbrain syndrome is the posterior commissure. It also remains possible, given the complexity of these lesions, that DBN may arise by different mechanisms in different patients.

### 4.3 Conclusions and future directions

DBN in association with the dorsal midbrain syndrome is rare, yet seems to be particularly associated with upgaze paresis in patients with congenital or acquired aqueductal stenosis. DBN due to posterior commissural disease may be the most likely anatomic localization and bilateral involvement of eye movement signals coursing through the posterior commissure may be a pre-requisite. In consideration of why this combination of findings is so rare, several possibilities arise: (1) it may be under-recognized given that DBN was subtle and present in central gaze only on fundoscopy in some of our patients; (2) perhaps non-human primates have a greater propensity to develop DBN with posterior commissural lesions due to up/down asymmetries in gaze control that may differ from humans, or (3) development of DBN requires a specific combination of prerequisite conditions that rarely co-occur in human disease. Future studies explicitly tracing posterior commissural projections and further detailed clinical correlations with quantitative ocular motor recordings will be invaluable in definitive understanding of the underlying mechanisms of DBN in the dorsal midbrain syndrome.

## Data Availability

The original contributions presented in the study are included in the article/supplementary material, further inquiries can be directed to the corresponding author.

## Supplementary material

SUPPLEMENTARY VIDEO S1 Downbeat nystagmus (DBN) in case 2. Initial portion of video in color was initial exam in clinic when DBN was clinically apparent with the patient fixating a visual target. Final portion of video in high resolution black and white was 2 months later, when low amplitude DBN was detected seated with the head pitched forward in the absence of visual fixation (see eye position traces in Figure 1D).

## Glossary

APF: accessory paraflocculus
BC: brachium conjunctivum (superior cerebellar peduncle)
BCd: BC decussation
CC: crus cerebri
CP: cerebral peduncles
III: oculomotor nucleus
IV: trochlear nucleus
VI: abducens nucleus
VIII: vestibular nucleus
XII: hypoglossal nucleus
F: flocculus
IC: inferior colliculus
INC: interstitial nucleus of Cajal
IO: inferior oblique
IR: inferior rectus
M: M group
MLF: medial longitudinal fasciculus
ND: nucleus of Darkschewitsch
NPC: nucleus of the posterior commissure
PAG: periaqueductal gray
PB: pineal body
PC: posterior commissure
PMT: paramedian tract cell groups
PTn: pretectal nucleus
RIMLF: rostral interstitial medial longitudinal fasciculus
RN: red nucleus
SC: superior colliculus
SN: substantia nigra
SO: superior oblique
SR: superior rectus
